# Examining the Independent Association Between Acculturative Stress and Psychological Distress Among Mexican Immigrants in New York City: An Exploratory Study

**DOI:** 10.1089/heq.2022.0137

**Published:** 2023-03-23

**Authors:** Sandra Verdaguer, Rachita Ramya, María Hernández, Karen R. Flórez

**Affiliations:** ^1^ Department of Community Health and Social Sciences, CUNY Graduate School of Public Health and Health Policy, New York, NY, USA.; ^2^ El Instituto: Institute of Latina/o, Caribbean and Latin America Studies of College of Liberal Arts and Science, University of Connecticut, Storrs, CT, USA.; ^3^ Environmental, Occupational and Geospatial Sciences Department, CUNY Graduate School of Public Health and Health Policy, New York, NY, USA.

**Keywords:** Mexican immigrants, acculturative stress, psychological distress

## Abstract

**Objective:**

This study examines the association between acculturative stress and psychological distress among Mexican immigrants living in New York City. It takes account factors such as language barriers, legal status, fear of deportation, and avoidance of social health and human services, and how these factors are implicated in the mental health status of the study population.

**Design:**

Study draws from a community-based sample of Mexican American adults from the Social Network of Mexican Americans study recruited from a church-based community center in the Bronx, New York. Eighty Mexican immigrants were included in this analysis. Descriptive statistics were used to display participants' characteristics. Pearson correlation and multiple regressions were run to determine the relationship between acculturative stress and psychological distress, and also with each of the items from the acculturative stress scale. Both scales have been validated among Spanish-speaking Latino immigrants.

**Results:**

A significant moderate positive relationship was found between acculturative stress and psychological distress. Within the acculturative stress scale, those items related to language discrimination, evasion of health services, and feeling guilty for leaving family/friends in home country had significant associations with increased psychological distress.

**Conclusion:**

The findings support the need for interventions that account for the major stressors associated with being a Mexican immigrant in the United States to prevent psychological distress, especially given the anti-immigration policies.

## Introduction

The burden of mental illnesses is particularly acute in the United States as its prevalence is the highest of all disease and it is the most common cause of disability.^1^ Chronic stress is one of the factors associated with mental disorders. Recent studies have found that chronic stress can modify the brain structure and these changes, along with other factors, can increase the probability of developing mental disorders.^2^

Mexican immigrants in the United States are disproportionately affected by chronic stress.^3^ They often face multiple stressors and contextual challenges when they migrate to the United States such as discrimination, isolation, marginalization, stigmatization, fear of deportation, exploitability, and victimization.^3^ The U.S. sociopolitical atmosphere may also negatively impact the psychological health of the immigrant community once they arrive in the United States.^4^ For example, the Personal Responsibility and Work Opportunity Reconciliation Act of 1996 banned immigrants entering the country legally from receiving federally funded benefits for their first 5 years of living in the United States.

This created a chilling effect for all public benefits for immigrants families in which affected care seeking and benefit enrollment even among those to whom the policy did not apply.^5–7^ A more recent example of this was the 2019 public charge rule in the Federal Register, which denied a path to citizenship to lawful immigrants who have received federally funded benefits, including noncash benefits such as Medicaid.^8^ Even though it has already been rescinded, it affected health care-seeking among immigrants even before the implementation due to the fear and confusion about the proposed 2019 public charge rule.^9^

Immigrants of Mexican descent have been particularly affected by these restrictive immigrant policies in different ways.^10–13^ For example, Almeida et al.^10^ found that Mexicans immigrants living in states with more restrictive immigration policies had higher levels of perceived discrimination than Mexicans immigrants living in states with less restrictive policies. Also, Aragones et al.^13^ found that Mexican immigrant families living in states with more restrictive insurance eligibility policies had lower rates of care access. These high levels of perceived discrimination and the barriers to health care access among Mexicans immigrants in states with more restrictive immigration policies may be detrimental to their mental health.

In fact, in another state-level analysis, Hatzenbuehler et al.^11^ found that Latinos in U.S. states with more restrictive immigration policies had higher rates of poor mental health days compared with Latinos in U.S. states with less restrictive immigration policies. As Morey^12^ discussed, anti-immigrant rhetorical and political actions lead to health disparities among immigrants' groups, undocumented persons, and people of color through increasing multilevel discrimination and stress, increasing detention and deportation, and limiting health resources.

Immigration to the United States, for some, is linked to socioeconomic disadvantages and unsafe housing, which increase chronic stress.^3^ Particularly, Mexican immigrants living in the United States are more likely to have low socioeconomic position and higher rates of unemployment and poverty compared with non-Hispanic whites and also compared with other Latino immigrants.^14^ For example, the median household income for Mexican immigrant families is $46,000, whereas for Central American immigrant and South American immigrant families are $48,200 and $60,000, respectively.^15^ A total of 18.6% of Mexican immigrants in the United States live in poverty compared with 18.3% of Central American immigrants and 11.7% of South American immigrants.^15^ The unemployment rate for Mexican immigrants is 4.2%, whereas for Central American immigrants and South American immigrants are 3.9% and 3.7%, respectively.^15^

All these aforementioned socioeconomic disadvantages are daily life chronic stressors that increase the risk for mental disorders over time. Among all mental disorders, psychological distress is a state of emotional suffering associated with stressors and demands that are difficult to cope with in daily life.^16^ Psychological distress is even more pronounced among undocumented immigrants because they are afraid of deportation and they tend to avoid social and human health services. This persistent fear and health care barriers serve as chronic stressors that then increase psychological distress among undocumented immigrants.^17^

According to the acculturation literature, acculturative stress plays an important role in the psychological well-being of immigrants.^18,19^ Acculturative stress comes from the result of cultural change, language learning and retention, separation from family, linguistic barriers, and adjustment to the new environment.^20^ Therefore, psychological distress could result as part of the acculturation stress, or the stressful process by which immigrants begin to espouse the norms, beliefs, and behaviors of the new host society.^21^ The literature suggests that acculturation levels could either increase or decrease acculturative stress.^22,23^ Being more acculturated can be not only associated with upward social mobility but also can increase exposure to and recognition of discrimination.^24,25^ Importantly, researchers are increasingly recognizing that this process does not occur in a vacuum and that its quality is likely shaped by broader social determinants that are fundamental to adaptation processes.^26,27^

For years, multiple studies have been conducted to understand the Latino health paradox as research has been shown that despite their vulnerability, the Latino population had lower-than-expected mortality rates and better birthing outcomes than non-Latinos.^28–30^ However, this health advantage seems to erode over time as a consequence of acculturation, dietary changes, decreased physical activity, poverty, and discrimination.^31^ Also, this pattern is not generalizable within or across Latino subgroups.

Latino population refers to individuals from the geographic region of Latin America, including Central America, South America, as well as the Caribbean. Despite some similarities within Latino population, there are significant differences between Mexicans and other Latino subgroups as they come from diverse social, economic, and geographic background.^32^ There is much diversity within the Latino population in their language, culture, customs, cuisine, art, and dance. Therefore, making a distinction is the first step to fully understand the Mexican community.

In terms of mental health, Escobar, Nervi, and Gara (2000) also found this Latino paradox in the Mexican-born immigrants, specifically. They conducted a study to compare the mental health status of Mexican-born immigrants and U.S.-born Mexican Americans. Their results showed that Mexican-born immigrants in the United States had better mental health status than U.S.-born Mexican Americans.^33^ However, when we analyze the mental health status of Mexican-born exclusively, the results showed that the prevalence of anxiety among Mexican-born people is higher among those who live in the United States than those who live in Mexico.^34^ In addition, this health advantage has not been found in studies conducted within Mexican-born immigrants. When comparing the psychological health of more recent Mexican immigrants versus more established Mexican immigrants, Gearing et al. (2021) found that more recent Mexican immigrants had significantly worse psychological health than more established Mexican immigrants.^35^

In a qualitative study, Paat and Green (2017) have documented several challenges and stressors that Mexican immigrants experienced while seeking legal services on the U.S.-Mexico border.^36^ However, little is known about the acculturation stressor factors that are implicated in the psychological distress of Mexican immigrants and in what degree they are associated. There is a need for identifying the specific stressors that acculturation and migration might have on the psychological distress of this community.

The rapidly growing Mexican population living in the United States requires public health research regarding the distinct acculturation stressors. There are about 11.2 million Mexican-born immigrants in the United States; the majority live in California (36%), Texas (22%), and Illinois (6%), with a sizable number in New York (1.9%).^37^ Mexican immigrants are a uniquely vulnerable subgroup of immigrants in the United States who have not received the appropriate attention in the literature.^38^ Most of the studies are limited to comparisons within the U.S. population, in which they compare Mexican Americans and non-Hispanic whites. Mexican immigrants face different stressors than those who are Mexican descent but born and raised in the United States.

It is necessary to account for this difference as there is a lack of studies that isolate the potential effect of acculturative stress on Mexican immigrants themselves.^39^ This study will fill this gap by examining acculturative stress and its association with the psychological distress of the Mexican immigrants living in New York City. We aim to get a better understanding of the acculturative stress factors and how these stressors are implicated in the mental health of this unique population. Guided by the integrative model of acculturation and salutogenesis in which differentiate integration, assimilation, separation, and marginalization as acculturation strategies,^40^ we decided to take account acculturative stress factors such as language barriers, legal status, fear of deportation, and evasion of social health and human services.

## Methods

### Data source

Data were obtained from the Social Network of Mexican Americans (SNMA) study, which was designed to explore the influence of the personal social networks of Mexican American adults on diabetes-related health behaviors by drawing on a conceptual framework that conceives acculturation as a two-level phenomenon involving the group and the individual. Eighty-one participants were recruited from a church-based community center in the Bronx. The church liaison introduced our research team to potential prayer and activity groups with large concentrations of Mexican American congregants.

She also identified potential participants through a telephone roster. Trained bilingual research assistants reached out to participants in person or through telephone to explain details of the study. People who were interested were screened. Eligibility criteria consisted of any person aged ≥18 years who self-identifies as Mexican, Mexican American, or Chicano. Only one participant was excluded from this analysis because they were born in the United States and, therefore, did not meet the inclusion criteria of being a Mexican immigrant to the United States.

Surveys were administered by bilingual researchers in either Spanish or English based on participants' preferences from January to June 2019. Written informed consent was obtained in a private space from all participants included in the study. Participants received a $15 gift card for completing the demographic/health survey. The SNMA study procedures were reviewed and approved by CUNY SPH Human Subjects Committee (IRB File No. 2018-1081).

### Measures

#### Psychological distress

Kessler Psychological Distress Scale (K-6) assessed participant's distress for a period of 30 days before administration of the test.^41^ K-6 involves six questions about a person's emotional state (e.g., “About how often during the past 30 days did you feel nervous? Hopeless? Restless or fidgety? So depressed that nothing could cheer you up? Everything was an effort? Worthless?”). Participants reported how often they have experienced depressive and anxiety symptoms within the past 30 days by rating K-6 items on a Likert-type scale that range from 0 (none of the time) to 4 (all of the time). Scores of the six questions were summed to yield an overall level of psychological distress, where greater scores correspond to higher levels of psychological distress.

Kessler et al. reported a Cronbach's alpha of 0.89 and it has been tested the successful use of the Spanish-language version of the K-6 in diverse racial population, including Mexican immigrants.^42,43^ Among our sample, the coefficient alpha for the K-6 also showed high reliability (0.797).^44^

#### Acculturative stress

Acculturative Stress Scale from the National Latino and Asian American Study was used in this study to measure the stress felt as a result of adapting one's own culture with a host culture.^45^ It is a nine-item scale with dichotomous responses (yes=1 or no=0) to the following questions: (1) feeling guilty for leaving family in a home country; (2) receiving the same level of respect that immigrants had in a home country; (3) limited contact with family and friends in a home country; (4) difficulties in interaction with others because of English proficiency; (5) being treated badly because of speaking English with an accent; (6) difficulties in finding work because of Latino descent; (7) being questioned about legal status; (8) concern about being deported if one were to go to a social or government agency; and (9) the avoidance of seeking health services due to fear of immigration officials.

All items were summed, with higher values representing higher acculturative stress. Item (2) was reverse scored for this analysis. Acculturative Stress Scale has been validated among Spanish-speaking Latino immigrants^46^ and a previous study in Latinos has found a Cronbach's alpha of 0.69.^47^ In our sample, the coefficient alpha for the Acculturative Stress Scale was exactly the same (0.69), which shows moderate reliability.^44^

#### Covariates

Established risk factors that contribute to psychological distress were controlled, including age, years of residency in the United States, gender, education level, employment status, household income, marital status, general health, and health insurance.^48–50^ Age, years of residency in the United States, education level, and household income were coded as continuous covariates and the rest as categorical covariates. Categorical covariates were included in the analysis as a set of dummy variables, such as gender (“male” vs. “female”), employment status (“employed” vs. “unemployed”), marital status (“not married and not living with partner” vs. “married or living with partner”), general health (“good, very good or excellent” vs. “fair or poor”), and health insurance status (“insured” vs. “uninsured”).

### Analysis plan

Before running the statistical analyses, K-6 and Acculturative Stress Scale were tested for normality and for internal consistency reliability. Descriptive statistics were used to display participants' characteristics.

Pearson correlation was performed to examine the association of acculturative stress and psychological distress. In addition, multiple regressions were run to determine the relationship between acculturative stress and psychological distress, and also with each of the items from the acculturative stress questionnaire, while controlling for age, years of residency in the United States, gender, education level, employment status, household income, marital status, general health, and health insurance. All statistical analyses were performed with IBM SPSS Statistics 25.

## Results

Tests of normality were performed to check for skewness and kurtosis of data distribution. As observed in Table 1, all the values fell within the commonly acceptable range of ±2 for skewness and kurtosis.^44,51,52^ Moreover, a visual observation of the normal Q–Q plots revealed that the K-6 and Acculturative Stress Scale were within the acceptable criteria for normality as the plots were fairly linear.

**Table 1.** Skewness and Kurtosis

	Skewness	Kurtosis
K-6	1.067	0.641
Acculturative Stress	0.460	−0.561

K-6, Kessler Psychological Distress Scale.

Table 2 presents demographic descriptive statistics.

**Table 2.** Descriptive Characteristics (*n*=80)

Years residency in United States, mean±SD	19.77±10.468
Years of age, mean±SD	43.13±11.379
Male, *n* (%)	24 (30)
Education level, *n* (%)	
<11th grade	47 (58)
High school graduate or GED	18 (22.5)
Some college and above	15 (18.8)
Employed, *n* (%)	53 (66.3)
Household income, *n* (%)	
<$10,000	20 (25.0)
$10,000–$29,999	38 (47.5)
≥$30,000	22 (27.5)
Marital status	
Not married and not living with partner	32 (40)
Married or living with partner	48 (60)
General health	
Good, very good, or excellent	38 (47.5)
Fair or poor	42 (52.5)
Health insurance	
Insured	46 (57.5)
Uninsured	34 (42.5)

GED, General Educational Development; SD, standard deviation.

The majority of the sample was female (*n*=56; 70%), employed (*n*=53; 66.3%), married (*n*=48; 60%), and had health insurance (*n*=46; 57.5%). Fair or poor self-rated overall health was common among the sample (*n*=42; 52.5%). The mean reported age was 43.13 years (standard deviation [SD]=11.4; range 20–68). Participants had lived, on average, 19.77 years in the United States (SD=10.47). Slightly greater than half (*n*=47, 58%) of the sample reported having 11 years or fewer of education; however, 22.5% (*n*=18) had a high school diploma or the General Educational Development, 18.8% (*n*=15) had attended at least 1 year of college or had a bachelor's degree or higher. A quarter of participants (*n*=20, 25%) reported an annual household income <$10,000; 47.5% (*n*=38) had a household income of $10,000–$29,999 and 27.5% (*n*=22) had a household income of ≥$30,000.

The Pearson correlation between acculturative stress and psychological distress of 0.429 indicates that there is a moderate positive relationship between the variables and this correlation is significant at the 0.01 level (*p*=0.000).

A multiple regression model was fitted to determine the association between acculturative stress and psychological distress while controlling for age, years of residency in the United States, gender, education level, employment status, household income, marital status, general health, and health insurance. The multiple regression model showed that with higher acculturative stress there is higher psychological distress (B coefficient=0.756; *p*=0.001), after controlling for confounders.

An additional regression was run to explore the relationship between each item of the acculturative stress scale and psychological distress after controlling for age, years of residency in the United States, gender, education level, employment status, household income, marital status, general health, and health insurance. Of the nine items of the Acculturative Stress Scale (Table 3), the statement of “feeling guilty for leaving family or friends in home country” was significantly associated with psychological distress (B coefficient=2.227; *p*=0.013).

**Table 3.** Relationship Between Each Item of the Acculturative Stress Scale and Psychological Distress

	B Coefficient^a^	p	95% CI
Feeling guilty for leaving family or friends in home country	2.227	0.013^*^	0.476 to 3.979
Not receiving the same level of respect that immigrants had in a home country	0.714	0.449	−1.155 to 2.583
Limited contact with family and friends in the home country	0.288	0.767	−1.644 to 2.220
Finding it hard interacting with others because of difficulties with English language	2.672	0.004^**^	0.884 to 4.460
Being treated badly because of poor English or speaking English with an accent	2.617	0.010^**^	0.656 to 4.577
Difficulties in finding work because of Latino descent	1.085	0.267	−0.849 to 3.018
Being questioned about legal status	0.901	0.350	−1.010 to 2.813
Concern about being deported if one were to go to a social or government agency	1.462	0.167	−0.624 to 3.548
Avoidance of seeking health services due to fear of immigration officials	2.491	0.032^*^	0.221 to 4.762

^a^ Controlling for age, years of residency in the United States, gender, education level, employment status, household income, marital status, general health, and health insurance.

^*^ Significance level of 0.05.

^**^ Significance level of 0.01.

CI, confidence interval.

Also, a significant relationship was found with the two items related to language discrimination “finding it hard interacting with others because of difficulties they have with the English language” (B coefficient=2.672; *p*=0.004) and “being treated badly because of their poor English or speaking English with an accent” (B coefficient=2.617; *p*=0.010). The item of “avoidance of seeking health services due to fear of immigration officials” was also significantly associated with psychological distress (B coefficient=2.491; *p*=0.032). All other five items were not significantly associated (*p*<0.05) with psychological distress, but they showed a positive relationship.

## Discussion

Our findings suggest a positive relationship between acculturative stress and psychological distress in the Mexican immigrant population living in New York City. They also highlight that not all aspects of acculturative stress are equally important in its relationship with psychological distress. That is, the subconstructs of fear of deportation and government bodies, stress from racial discrimination as well as stress associated with immigrating and adapting to a new environment are particularly salient for this community.

Other studies have shown that fear of government bodies and feeling of mistrust in authority prevents immigrants from seeking social and health services.^53–55^ The findings of these studies suggest that evasiveness of health services is directly associated with immigrant status and that psychological distress is influenced by fear of family separation and deportation. Our findings are congruent with other studies conducted with immigrants that discuss the direct impact of legal status on the psychological distress of the immigrant community^56–58^ and adds evidence about Mexican immigrants and the association between avoidance of care-seeking and psychological distress. This finding emphasizes the need for immigration and health care policies that do not discourage Mexican-born immigrants from receiving health care services.

Our finding that language barriers when communicating in English and discriminatory practices as a result of said language barriers are associated with greater psychological distress highlight the need to further understand how microagressions affect the acculturation process. For example, microaggressions and discrimination could result in feelings of being “othered”^59^ and create psychological barriers toward fully adopting English. Indeed, higher life satisfaction and resilience in individuals who speak both English and Spanish have been found compared with their monolingual Spanish-speaking counterparts.^60^

If this feeling of “being othered” could happen in a cosmopolitan and diverse city such as New York City, which has attracted more Mexican immigrant in the past decades, one can conceive that this feeling of “being othered” might be even more pronounced in less diverse areas to which Mexican immigrants are currently migrating, such as in the southern and central regions of the United States.^61^ Studies among Mexican immigrants settling in new destinations are needed to understand the potential impact of “being othered” and the relationship between language barriers and psychological distress.

Our finding that guilt related to leaving a family member back home was significantly associated with psychological distress should be viewed within the broader research that has shown migration often results in family separation and instability.^62–65^ Furthermore, this research shows that a particularly salient mechanism between migration-related separation and psychological distress may be through family cohesion. For example, one study that specifically looked at the relationship between psychological distress and family cohesion in the Mexican American population using the K-10 found that greater psychological distress was statistically associated with higher levels of family cultural conflict.^63^

However, there seems to be a greater emphasis on the positive aspects of family cohesion among Latinos in health research, given the cultural trait broadly defined as familism, or the cultural value that emphasizes close supportive family relationships and prioritizing family over the self.^66^ Future research should assess the protective effect of traditional family networks for the association between acculturation stress and psychological distress in Mexican immigrants.

Taken together, our findings are consistent with the burgeoning research that examines psychological distress among Latino immigrants. For example, a recent study examining the interpersonal, cultural, and individual determinants of psychological distress among young adult Latina women who immigrated recently to the United States found that higher levels of psychological distress were associated with acculturative stress and undocumented immigration status.^67^ Another study with young Latina immigrants found that higher levels of acculturative stress were associated with greater psychological distress when the participants indicated more negative religious coping.^68^ Religion is an essential source of strength in the Mexican culture and future research could explore the use of religious beliefs and practices to cope with acculturation stressors and how this is associated with psychological distress in Mexican immigrants.

### Limitations

Owing to the cross-sectional nature of the study design, the causality and temporality of the relationship between acculturative stress and psychological distress found cannot be determined. All data were self-reported and vulnerable to recall and social desirability biases. Other concerns within this study are the small sample size and the possibility of self-selection bias. The findings could also be bound by location and time, primarily reflective of low-income neighborhood Mexican communities living in the Bronx and pose questions on generalizability. Despite the limitations, the study offers insight into the effects of acculturative stress on the Mexican American population living in New York City. Also, our study used reliable exposure and outcome measures such as the Acculturative Stress Scale and the K-6.

## Health Equity Implications

This study found a significant positive relationship between acculturative stress and psychological distress in Mexican immigrants, even after controlling for several covariates. Within the acculturative stress scale, those items related to language discrimination, evasion of health services due to fear of immigration officials, and the guilty feeling for leaving family/friends in home country had more powerful and harmful association with psychological distress.

This study sheds light on the health effect of macro-level factors such as social structures, racial hierarchies, economic inequalities, and citizenship status on immigrant communities.^27^ The study findings have implications for policy-related changes to address language barriers, discriminatory practices, and urban conflicts faced by Mexican immigrants due to intercultural tensions. There is a need toward a more conceptual model that locates the discriminatory practices faced by Mexican immigrants living in the United States.^69^

Future studies could be done in this regard to primarily address the structural barriers in immigrant psychological distress. A lot of the studies about the mental health status in the Mexican American population are not capturing the chronic stressors related to the immigration/acculturation experience and, therefore, they are not including the stressors that the Mexican immigrant population experience. In addition, a strength-based research approach, including increasing the focus on resilience in Mexican immigrant communities, could also be a potential direction.

In terms of community-based approaches, these findings can help health care providers and immigrant social workers to increase their awareness on the necessity of culturally appropriate services for Mexican immigrants to overcome the negative impact of acculturative stress. Culturally appropriate interventions could include supportive coaching sessions to address acculturative stressors.

## Abbreviations Used

CI: confidence interval
K-6: Kessler Psychological Distress Scale
SD: standard deviation
SNMA: Social Network of Mexican Americans

## References

[1] Centers for Disease Control and Prevention. About Mental Health. 2021. Available from: https://www.cdc.gov/mentalhealth/learn/index.htm [Last accessed: September 29, 2021].

[2] Chetty S, Friedman AR, Taravosh-Lahn K, et al. Stress and glucocorticoids promote oligodendrogenesis in the adult hippocampus. Mol Psychiatry 2014;19(12):1275–1283; doi: 10.1038/mp.2013.190 24514565 PMC4128957

[3] Garcini LM, Galvan T, Malcarne V, et al. Mental disorders among undocumented Mexican immigrants in high-risk neighborhoods: Prevalence, comorbidity, and vulnerabilities. J Consult Clin Psychol 2017;85(10):927–936; doi: 10.1037/ccp0000237 28956948 PMC6049826

[4] Martinez O, Wu E, Sandfort T, et al. Evaluating the impact of immigration policies on health status among undocumented immigrants: A systematic review. J Immigr Minor Health 2015;17(3):947–970; doi: 10.1007/s10903-013-9968-4 24375382 PMC4074451

[5] Derose KP, Escarce JJ, Lurie N. Immigrants and health care: Sources of vulnerability. Health Aff (Millwood) 2007;26(5):1258–1268; doi: 10.1377/hlthaff.26.5.1258 17848435

[6] Derose KP, Bahney BW, Lurie N, et al. Review: Immigrants and health care access, quality, and cost. Med Care Res Rev 2009;66(4):355–408; doi: 10.1177/1077558708330425 19179539

[7] Hagan J, Rodriguez N, Capps R, et al. The effects of recent welfare and immigration reforms on immigrants' access to health care. Int Migrat Rev 2003;37(2):444–463.

[8] Khullar D, Chokshi DA. Challenges for immigrant health in the USA—The road to crisis. Lancet 2019;393(10186):2168–2174; doi: 10.1016/S0140-6736(19)30035-2 30981536

[9] Bernstein H, McTarnaghan S, Gonzalez D. Safety Net Access in the Context of the Public Charge Rule. 2019. Available from: https://www.urban.org/research/publication/safety-net-access-context-public-charge-rule [Last accessed: December 9, 2021].

[10] Almeida J, Biello KB, Pedraza F, et al. The association between anti-immigrant policies and perceived discrimination among Latinos in the US: A multilevel analysis. SSM Popul Health 2016;2:897–903; doi: 10.1016/j.ssmph.2016.11.003 29349196 PMC5757908

[11] Hatzenbuehler ML, Prins SJ, Flake M, et al. Immigration policies and mental health morbidity among Latinos: A state-level analysis. Soc Sci Med 2017;174:169–178; doi: 10.1016/j.socscimed.2016.11.040 28043019 PMC5258771

[12] Morey BN. Mechanisms by which anti-immigrant stigma exacerbates racial/ethnic health disparities. Am J Public Health 2018;108(4):460–463; doi: 10.2105/AJPH.2017.304266 29470116 PMC5846442

[13] Aragones A, Zamore C, Moya EM, et al. The impact of restrictive policies on Mexican immigrant parents and their children's access to health care. Health Equity 2021;5(1):612–618; doi: 10.1089/heq.2020.0111 34909528 PMC8665780

[14] Orrenius P, Zavodny M. Trends in poverty and inequality among Hispanics. WP 2011;2011:1109; doi: 10.24149/wp1109

[15] Pew Research Center. Immigrants in America: Current Data and Demographics. 2020. Available from: https://www.pewresearch.org/hispanic/2020/08/20/facts-on-u-s-immigrants-current-data/ [Last accessed: October 28, 2021].

[16] Arvidsdotter T, Marklund B, Kylén S, et al. Understanding persons with psychological distress in primary health care. Scand J Caring Sci 2016;30(4):687–694; doi: 10.1111/scs.12289 26463897

[17] Hacker K, Anies M, Folb BL, et al. Barriers to health care for undocumented immigrants: A literature review. Risk Manag Healthc Policy 2015;8:175–183; doi: 10.2147/RMHP.S70173 26586971 PMC4634824

[18] Serafica R, Lekhak N, Bhatta T. Acculturation, acculturative stress and resilience among older immigrants in United States. Int Nurs Rev 2019;66(3):442–448; doi: 10.1111/inr.12522 31106411

[19] Bernal DR, Misiaszek KH, Ayala J, et al. Second-class citizens? Subjective social status, acculturative stress, and immigrant well-being. SN Soc Sci 2022;2(7):96; doi: 10.1007/s43545-022-00371-2

[20] Derr AS. Mental health service use among immigrants in the United States: A systematic review. Psychiatr Serv 2016;67(3):265–274; doi: 10.1176/appi.ps.201500004 26695493 PMC5122453

[21] Abraído-Lanza AF, Echeverría SE, Flórez KR. Latino immigrants, acculturation, and health: Promising new directions in research. Annu Rev Public Health 2016;37:219–236; doi: 10.1146/annurev-publhealth-032315-021545 26735431 PMC5337110

[22] Potochnick SR, Perreira KM. Depression and anxiety among first-generation immigrant Latino youth: Key correlates and implications for future research. J Nerv Ment Dis 2010;198(7):470–477; doi: 10.1097/NMD.0b013e3181e4ce24 20611049 PMC3139460

[23] Saadi A, Ponce NA. Worse mental health among more-acculturated and younger immigrants experiencing discrimination: California Health Interview Survey, 2015–2016. J Gen Intern Med 2020;35(5):1419–1426; doi: 10.1007/s11606-019-05412-w 31677103 PMC7210364

[24] Torres L, Driscoll MW, Voell M. Discrimination, acculturation, acculturative stress, and Latino psychological distress: A moderated mediational model. Cultur Divers Ethnic Minor Psychol 2012;18(1):17–25; doi: 10.1037/a0026710 22250895 PMC3340887

[25] Lee S, O'Neill AH, Ihara ES, et al. Change in self-reported health status among immigrants in the United States: Associations with measures of acculturation. PLoS One 2013;8(10):e76494; doi: 10.1371/journal.pone.0076494 24098515 PMC3788132

[26] Abraído-Lanza AF, Armbrister AN, Flórez KR, et al. Toward a theory-driven model of acculturation in public health research. Am J Public Health 2006;96(8):1342–1346; doi: 10.2105/AJPH.2005.064980 16809597 PMC1522104

[27] Castañeda H, Holmes SM, Madrigal DS, et al. Immigration as a social determinant of health. Annu Rev Public Health 2015;36:375–392; doi: 10.1146/annurev-publhealth-032013-182419 25494053

[28] Castro FG. Emerging Hispanic Health Paradoxes. Am J Public Health 2013;103(9):1541; doi: 10.2105/AJPH.2013.301529 23865708 PMC3780698

[29] Ruiz JM, Steffen P, Smith TB. Hispanic Mortality Paradox: A systematic review and meta-analysis of the longitudinal literature. Am J Public Health 2013;103(3):e52–e60; doi: 10.2105/AJPH.2012.301103 PMC3673509 23327278

[30] Velasco-Mondragon E, Jimenez A, Palladino-Davis AG, et al. Hispanic health in the USA: A scoping review of the literature. Public Health Rev 2016;37:31; doi: 10.1186/s40985-016-0043-2 29450072 PMC5809877

[31] Teruya SA, Bazargan-Hejazi S. The Immigrant and Hispanic Paradoxes: A systematic review of their predictions and effects. Hisp J Behav Sci 2013;35(4):486–509; doi: 10.1177/0739986313499004 26120244 PMC4478591

[32] González Burchard E, Borrell LN, Choudhry S, et al. Latino Populations: A unique opportunity for the study of race, genetics, and social environment in epidemiological research. Am J Public Health 2005;95(12):2161–2168; doi: 10.2105/AJPH.2005.068668 16257940 PMC1449501

[33] Escobar JI, Nervi CH, Gara MA. Immigration and mental health: Mexican Americans in the United States. Harv Rev Psychiatry 2000;8(2):64–72; doi: 10.1080/hrp_8.2.64 10902095

[34] Borges G, Zamora B, García J, et al. Symptoms of anxiety on both sides of the US-Mexico border: The role of immigration. J Psychiatr Res 2015;61:46–51; doi: 10.1016/j.jpsychires.2014.12.004 25543519 PMC4308435

[35] Gearing RE, Washburn M, Torres LR, et al. Immigration policy changes and the mental health of Mexican-American immigrants. J Racial Ethn Health Disparities 2021;8(3):579–588; doi:10.1007/s40615-020-00816-5 32661921

[36] Paat YF, Green R. Mental health of immigrants and refugees seeking legal services on the US-Mexico border. Transcult Psychiatry 2017;54(5-6):783–805; doi:10.1177/1363461517746316 29226794

[37] Migration Policy Institute. U.S. Immigrant Population by State and County. 2019. Available from: https://www.migrationpolicy.org/programs/data-hub/charts/us-immigrant-population-state-and-county [Last accessed: December 21, 2022].

[38] Breslau J, Borges G. Editorial: Immigration and mental health in modern societies. Front Psychiatry 2020;11:1083; doi: 10.3389/fpsyt.2020.600763 PMC7641944 33192743

[39] Breslau J, Borges G, Tancredi D, et al. Migration from Mexico to the US and subsequent risk for depressive and anxiety disorders: A cross-national study. Arch Gen Psychiatry 2011;68(4):428–433; doi: 10.1001/archgenpsychiatry.2011.21 21464367 PMC3733092

[40] Riedel J, Wiesmann U, Hannich H-J. An integrative theoretical framework of acculturation and salutogenesis. Internat Rev Psychiatry 2011;23(6):555–564; doi: 10.3109/09540261.2011.637912 22272594

[41] Kessler RC, Barker PR, Colpe LJ, et al. Screening for serious mental illness in the general population. Arch Gen Psychiatry 2003;60(2):184–189; doi: 10.1001/archpsyc.60.2.184 12578436

[42] Kessler RC, Andrews G, Colpe LJ, et al. Short screening scales to monitor population prevalences and trends in non-specific psychological distress. Psychol Med 2002;32(6):959–976; doi: 10.1017/s0033291702006074 12214795

[43] Kessler RC, Green JG, Gruber MJ, et al. Screening for serious mental illness in the general population with the K6 screening scale: Results from the WHO World Mental Health (WMH) survey initiative. Int J Methods Psychiatr Res 2010;19(Suppl. 1):4–22; doi: 10.1002/mpr.310 20527002 PMC3659799

[44] Hinton PR, McMurray I, Brownlow C. SPSS Explained. 1st ed. Routledge: London; 2004.

[45] Alegria M, Takeuchi D, Canino G, et al. Considering context, place and culture: The National Latino and Asian American Study. Int J Methods Psychiatr Res 2006;13(4):208–220; doi: 10.1002/mpr.178 PMC2774128 15719529

[46] Alegria M, Vila D, Woo M, et al. Cultural relevance and equivalence in the NLAAS instrument: Integrating etic and emic in the development of cross-cultural measures for a psychiatric epidemiology and services study of Latinos. Int J Methods Psychiatr Res 2006;13(4):270–288; doi: 10.1002/mpr.181 PMC2771729 15719532

[47] DeVylder JE, Oh HY, Yang LH, et al. Acculturative stress and psychotic-like experiences among Asian and Latino immigrants to the United States. Schizophr Res 2013;150(1):223–228; doi: 10.1016/j.schres.2013.07.040 23932446 PMC3896050

[48] Jurado D, Alarcón RD, Martínez-Ortega JM, et al. Factors associated with psychological distress or common mental disorders in migrant populations across the world. Rev Psiquiatr Salud Ment 2017;10(1):45–58; doi: 10.1016/j.rpsmen.2017.02.004 27291831

[49] Ritsner M, Ponizovsky A, Nechamkin Y, et al. Gender differences in psychosocial risk factors for psychological distress among immigrants. Compr Psychiatry 2001;42(2):151–160; doi: 10.1053/comp.2001.19750 11244152

[50] Ward B, Martinez M. Health insurance status and psychological distress among US adults aged 18–64 years. Stress Health J Int Soc Invest Stress 2014;31(4):324–335; doi: 10.1002/smi.2559 PMC4658514 24403273

[51] George D, Mallery P. SPSS for Windows Step by Step: A Simple Guide and Reference, 17.0 Update. Allyn & Bacon: Boston, MA, USA; 2010.

[52] Gravetter FJ, Wallnau LB, Forzano L-AB. Essentials of Statistics for the Behavioral Sciences. 9th ed. Cengage Learning: Belmont, CA, USA; 2017.

[53] Arbona C, Olvera N, Rodriguez N, et al. Acculturative stress among documented and undocumented Latino immigrants in the United States. Hisp J Behav Sci 2010;32(3):362–384; doi: 10.1177/0739986310373210 25484488 PMC4254683

[54] Callaghan T, Washburn DJ, Nimmons K, et al. Immigrant health access in Texas: Policy, rhetoric, and fear in the Trump era. BMC Health Serv Res 2019;19(1):342; doi: 10.1186/s12913-019-4167-1 31164114 PMC6549327

[55] Kerani RP, Kwakwa HA. Scaring undocumented immigrants is detrimental to public health. Am J Public Health 2018;108(9):1165–1166; doi: 10.2105/AJPH.2018.304596 30089000 PMC6085039

[56] McGuire S, Martin K. Fractured migrant families: Paradoxes of hope and devastation. Fam Community Health 2007;30(3):178–188; doi: 10.1097/01.FCH.0000277761.31913.f3 17563480

[57] Suârez-Orozco C, Todorova ILG, Louie J. Making up for lost time: The experience of separation and reunification among immigrant families. Fam Process 2002;41(4):625–643; doi: 10.1111/j.1545-5300.2002.00625.x 12613121

[58] Sullivan MM, Rehm R. Mental health of undocumented Mexican immigrants: A review of the literature. ANS Adv Nurs Sci 2005;28(3):240–251; doi: 10.1097/00012272-200507000-00006 16106153

[59] Viruell-Fuentes EA. Beyond acculturation: Immigration, discrimination, and health research among Mexicans in the United States. Soc Sci Med 2007;65(7):1524–1535; doi: 10.1016/j.socscimed.2007.05.010 17602812

[60] Marsiglia FF, Booth JM, Baldwin A, et al. Acculturation and life satisfaction among immigrant Mexican adults. Adv Soc Work 2013;14(1):49–64. 24403865 PMC3881437

[61] Terrazas ATA. Immigrants in New-Destination States. 2011. Available from: https://www.migrationpolicy.org/article/immigrants-new-destination-states [Last accessed: December 1, 2021].

[62] Miller A, Hess JM, Bybee D, et al. Understanding the mental health consequences of family separation for refugees: Implications for policy and practice. Am J Orthopsychiatry 2018;88(1):26–37; doi: 10.1037/ort0000272 28617002 PMC5732089

[63] Rivera FI, Guarnaccia PJ, Mulvaney-Day N, et al. Family cohesion and its relationship to psychological distress among Latino groups. Hisp J Behav Sci 2008;30(3):357–378; doi: 10.1177/0739986308318713 19444326 PMC2681258

[64] Roche KM, Vaquera E, White RMB, et al. Impacts of immigration actions and news and the psychological distress of US Latino parents raising adolescents. J Adolesc Health 2018;62(5):525–531; doi: 10.1016/j.jadohealth.2018.01.004 29503033 PMC5930061

[65] Rojas-Flores L, Clements ML, Hwang Koo J, et al. Trauma and psychological distress in Latino citizen children following parental detention and deportation. Psychol Trauma 2017;9(3):352–361; doi: 10.1037/tra0000177 27504961

[66] Sabogal F, Marín G, Otero-Sabogal R, et al. Hispanic familism and acculturation: What changes and what doesn't? Hisp J Behav Sci 1987;9(4):397–412; doi: 10.1177/07399863870094003

[67] Dillon FR, Ertl MM, Verile M, et al. A social ecological study of psychological distress among recently immigrated, Latina young adults. J Lat Psychol 2018;7(1):39–58; doi: 10.1037/lat0000106 30800533 PMC6386459

[68] Silva ND, Dillon FR, Verdejo TR, et al. Acculturative stress, psychological distress, and religious coping among Latina young adult immigrants. Counsel Psychol 2017;45(2):213–236; doi: 10.1177/0011000017692111 PMC5636182 29033462

[69] Pérez-Soria J. Mexican immigrants in the United States: A review of the literature on integration, segregation and discrimination. Estud fronterizos 2017;18(37):1–17; doi: 10.21670/ref.2017.37.a01

